# The Impact of a Pre-Dialysis Educational Program on the Mode of Renal Replacement Therapy in a Saudi Hospital: A Retrospective Cohort Study

**DOI:** 10.7759/cureus.11981

**Published:** 2020-12-08

**Authors:** Ahlam A Alghamdi, Khalid A Almotairy, Roqaya Moeedh Aljoaid, Nedaa Anwar Al Turkistani, Rawan Walid Domyati, Morsy Mohamed Morsy Abdelrahman, Kholod Samer Shobain, Cathariena M Uys

**Affiliations:** 1 Health Education Department, King Fahad Armed Forces Hospital, Jeddah, SAU; 2 Family Medicine: Health Education Department, King Fahad Armed Forces Hospital, Jeddah, SAU; 3 Nephrology Department, King Fahad Armed Forces Hospital, Jeddah, SAU; 4 Nursing: Quality Department, King Fahad Armed Forces Hospital, Jeddah, SAU

**Keywords:** peritoneal dialysis, hemodialysis, end-stage renal disease, pre-dialysis education

## Abstract

Background

Self-care and peritoneal dialysis (PD) benefits have been underutilized in patients with end-stage renal disease (ESRD). The pre-dialysis education program (PDEP) has been generally introduced as an acceptable tool in increasing the rates of PD and has been reportedly recommended for ESRD patients as part of the introduced care. We aim to study the effect of PDEP on ESRD and whether they would prefer PD of center-based hemodialysis (HD).

Methods

This is a retrospective cohort study that was done at King Fahad Armed Forces Hospital in Jeddah, Saudi Arabia, in the dialysis center. Data were collected on patients and included demographics, preference of renal replacement therapy modality, and other possible factors that may affect patient choices such as educational level, economic status, and age.

Results

A total of 213 ESRD patients that met our criteria were included, with a total of 75 patients receiving PDEP. Out of those who received the PDEP, 57.3% and 42.7% of patients decided to perform HD and PD, respectively. There was a significant impact of PDEP on reducing HD choice [OR (95% CI) = 0.11 (0.05-0.24); P-value < 0.001]. Infections did not occur in 50.5% of the included patients while 45.8%, 3.3%, and 0.5% had central line-associated bloodstream infection (CLABSI), other infections, and peritonitis, respectively. Most of the PD patients (81.8%) did not have an infection as compared to 42.3% of the HD patients. HD was also associated with increased admission days [OR (95% CI) = 1.27 (1.07-1.51); P-value = 0.007].

Conclusion

We found that PDEP positively impacted the rate of PD while PD was associated with favorable outcomes and lower infection rates, emphasizing the importance of an educational program.

## Introduction

Incidence rates are growing and have reached up to 6%-8% in end-stage renal disease (ESRD) globally [1]. In the Kingdom of Saudi Arabia (KSA), the rate has been also reportedly increasing, as the Saudi Center for Organ Transplantation (SCOT) previously estimated that 19,659 patients underwent either hemodialysis (HD) or peritoneal dialysis (PD), with the largest proportion favoring HD (18,270 patients) in 2017 [2-3]. Favoring either of the renal replacement therapy (RRT) modalities is hugely variable among the different healthcare systems managing ESRD patients worldwide. Many factors have been involved in this preference and are usually non-medical. For example, the availability of resources and trained personnel, bias from physicians, financial aspects, and patients’ cultures and habits may influence the choice of one modality over the other [4].

When comparing hospital-based HD to self-dialysis, which includes PD and home-based HD, self-care dialysis has been associated with better outcomes regarding patients’ quality of life (QoL) in addition to huge reductions concerning financial burdens [5-7]. Previous reports usually referred to PD as the only self-care dialysis modality, with reported favorable outcomes [8-11]. Although many investigations have previously reported that the potential use and benefit from PD are generally underestimated, many benefits have been reported regarding its early use in an integrated way with other modalities [12-14]. The low rate for using PD is probably due to structural obstacles, nephrologists' attitudes, together with the reduced awareness of chronic kidney disease (CKD) patients towards this modality [15-16]. Therefore, boosting patients’ awareness of this mode can lead to an increased prevalence of patients undergoing PD.

The pre-dialysis education program (PDEP) has been generally introduced as an acceptable tool in this field and has been reportedly recommended for ESRD patients as part of the introduced care. The application of this program leads to delivering informative data to the patients and increases their awareness of the most suitable modality to follow, recommendations about lifestyle modifications, and the best treatment regimen to follow. Moreover, many investigations have reported that applying PDEP was generally associated with lower mortality rates by reducing the need to conduct urgent dialysis because of a higher awareness status of patients [17]. Although many studies have demonstrated the efficacy of PDEP on increasing patients’ awareness [9-10], many factors related to patients’ preference and selection of the best modality based on their QoL are still vague. In this study, we aim to study the impact of conducting PDEP on patients’ choice regarding the preference of using PD over HD and the determinants of such preference.

## Materials and methods

Study design

This is a retrospective cohort study that was conducted using the data of all patients who started renal replacement therapy either by hemodialysis or peritoneal dialysis and were part of the renal education program in King Fahad Armed Forces Hospital in Jeddah, Saudi Arabia. The data included all patients one year before and one year after the start of the PDEP in March 2019. The educational program was constructed according to the Bagnis et al. program that was published in 2015 [18].

Study population

The rate at which patients chose peritoneal dialysis in ESRD patients who received PDEP was compared to those who did not receive PDEP over a period of two years (one year before and one year after the start of PDEP. All patients with complete files were included in the study. Meanwhile, patients who started renal replacement therapy outside our hospital or those with an emergency, unplanned initiation of hemodialysis were excluded. A comparison of baseline characteristics between the patient cohorts prior to the introduction of the education program and the group after implementation was illustrated to assess matching.

Data collection tool

Data were collected utilizing a pre-designed data collection form. The collected data included patient demographic data, choice of renal replacement therapy modality, educational level, economical status, and age. The possible confounding bias was dealt with using an appropriate statistical analysis through multivariate analysis. The source of the data was the patients’ medical files which were available for all health educators. Any missing data were acquired through a telephone call or a direct interview with the patient when possible.

Statistical analysis

All data were analyzed using R software version 4.0.2 [19] using the packages (Rcmdr) [20] and (glm2) [21]. We represent nominal variables as frequencies and percentages by conducting the chi-square test (or Fisher’s exact test), as appropriate, to test the difference, according to the presence or absence of PDEP. Furthermore, we used multivariate logistic regression to identify any possible association between PDEP and choice of dialysis modality [22]. In addition, we measured the impact of dialysis modality on patient outcomes [22]. In the multivariate model, the data were adjusted for patients' age, gender, socioeconomic status, education, and the personnel providing the program. Regression results were expressed as odds ratios (ORs) and 95% confidence interval (95% CI). For all statistical tests, P-value < 0.05 was considered statistically significant.

## Results

Baseline sociodemographic characteristics

**Figure 1:**
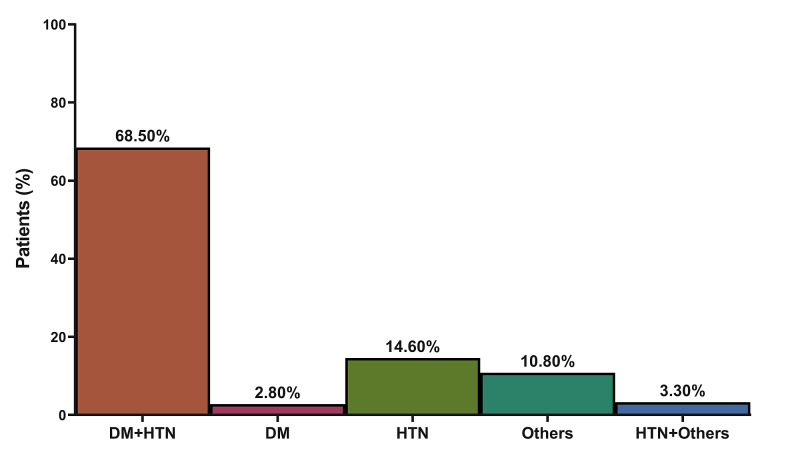
Distribution of end-stage renal disease causes among the included patients DM: diabetes mellitus; HTN: hypertension

A total of 213 patients were included in the current study; out of them, 35.2% were provided with PDEP and the other 64.8% were not. Nearly half of the patients (46.9%) were greater than 61 years old and 43.7% of them were males while most of them were either coming from a low-class (45.1%) or middle-class (48.8%) socioeconomic status. The majority of the patients (94.3%) earned 5,000 or more Saudi Riyals per month and most of them were residing in Jeddah City (84.0%). Regarding the educational level among the included patients, 12.2% had a college degree or higher, 46.9% had a high school degree or less, and 40.8% were illiterate. Half of the patients (49.8%) were included in the educational program with no referring personnel while 34.7% were referred by a doctor, 14.6% were referred by a health educator, and 0.9% were referred by a nurse. Furthermore, there was a statically significant difference in patients of PDEP when compared to non-PDEP, in terms of educational status (P-value = 0.036), presence of a caregiver (P-value = 0.03), and referral personnel (P-value < 0.001) (Table 1). The causes of ESRD were variable; some patients had one single cause while some had a combination of causes (Figure 1). A combination of diabetes mellitus (DM) and hypertension (HTN) was the most common cause of ESRD (68.5%). This was followed by HTN (14.6%), other conditions (10.8%), and DM (2.8%).

**Table 1:** Characteristics of the included studies * statistically significant, N: numbers

Variables		Pre-dialysis education program						P-values	
		Yes N=75 (35.2%)		No N= 138 (64.8%)		Total N= 213 (100)			
		N	%	N	%	N	%		
Patient age (Years)	Less than 12	0	0.0	4	2.9	4	1.9	0.24	
	12-41 years	18	24.0	21	15.2	39	18.3		
	40-61 years	24	32.0	46	33.3	70	32.9		
	More 61	33	44.0	67	48.6	100	46.9		
Gender	Female	39	52.0	81	58.7	120	56.3	0.347	
	Male	36	48.0	57	41.3	93	43.7		
Socioeconomic status	High class	5	6.7	8	5.8	13	6.1	0.548	
	Middle class	40	53.3	64	46.4	104	48.8		
	Low class	30	40.0	66	47.8	96	45.1		
Income (Saudi Riyals)	Less than 5000 SR.	30	40.0	67	48.6	97	45.5	0.477	
	5000-15000 SR.	40	53.3	64	46.4	104	48.8		
	More than 15000 SR.	5	6.7	7	5.1	12	5.6		
Residential area	In Jeddah	67	89.3	112	81.2	179	84.0	0.12	
	Outside Jeddah	8	10.7	26	18.8	34	16.0		
Educational status	Illiterate	27	36.0	60	43.5	87	40.8	0.036*	
	High school or less	33	44.0	67	48.6	100	46.9		
	College or higher	15	20.0	11	8.0	26	12.2		
Presence of a caregiver	No	26	34.7	29	21.0	55	25.8	0.03*	
	Yes	49	65.3	109	79.0	158	74.2		
Referred by	Doctor	55	73.3	19	13.8	74	34.7	< 0.001*	
	Nurse	0	0.0	2	1.4	2	0.9		
	Health Educator	20	26.7	11	8.0	31	14.6		
	None	0	0.0	106	76.8	106	49.8		

Pre-dialysis education and renal replacement therapy mode

The PDEP was introduced to 75 patients at the beginning of the program; out of those, 57.3% decided to perform hemodialysis and 42.7% decided to go with the peritoneal dialysis option. Overall, HD was performed in 168 patients as compared to 45 ones with PD. The introduction of the PDEP was associated with a significant reduction in HD rates [OR (95% CI) = 0.11 (0.05-0.24); P-value < 0.001] (Table 2).

**Table 2:** The impact of pre-dialysis education on the mode of renal replacement therapy * Statistically significant; ¶ Effect of Pre-dialysis education on the odds of choosing peritoneal dialysis, where the data were adjusted for patients age, gender, socioeconomic status, and education. N: numbers, OR: odds ratio

Variable			Pre-dialysis education program				Multivariate logistic regression¶		
			Yes		No		OR (95 CI)		P-value
			N	%	N	%			
Mode of dialysis	Hemodialysis	43		57.3	125	90.6	0.11 (0.05-0.24)	< 0.001*	
	Peritoneal dialysis	32		42.7	13	9.4			

**Figure 2:**
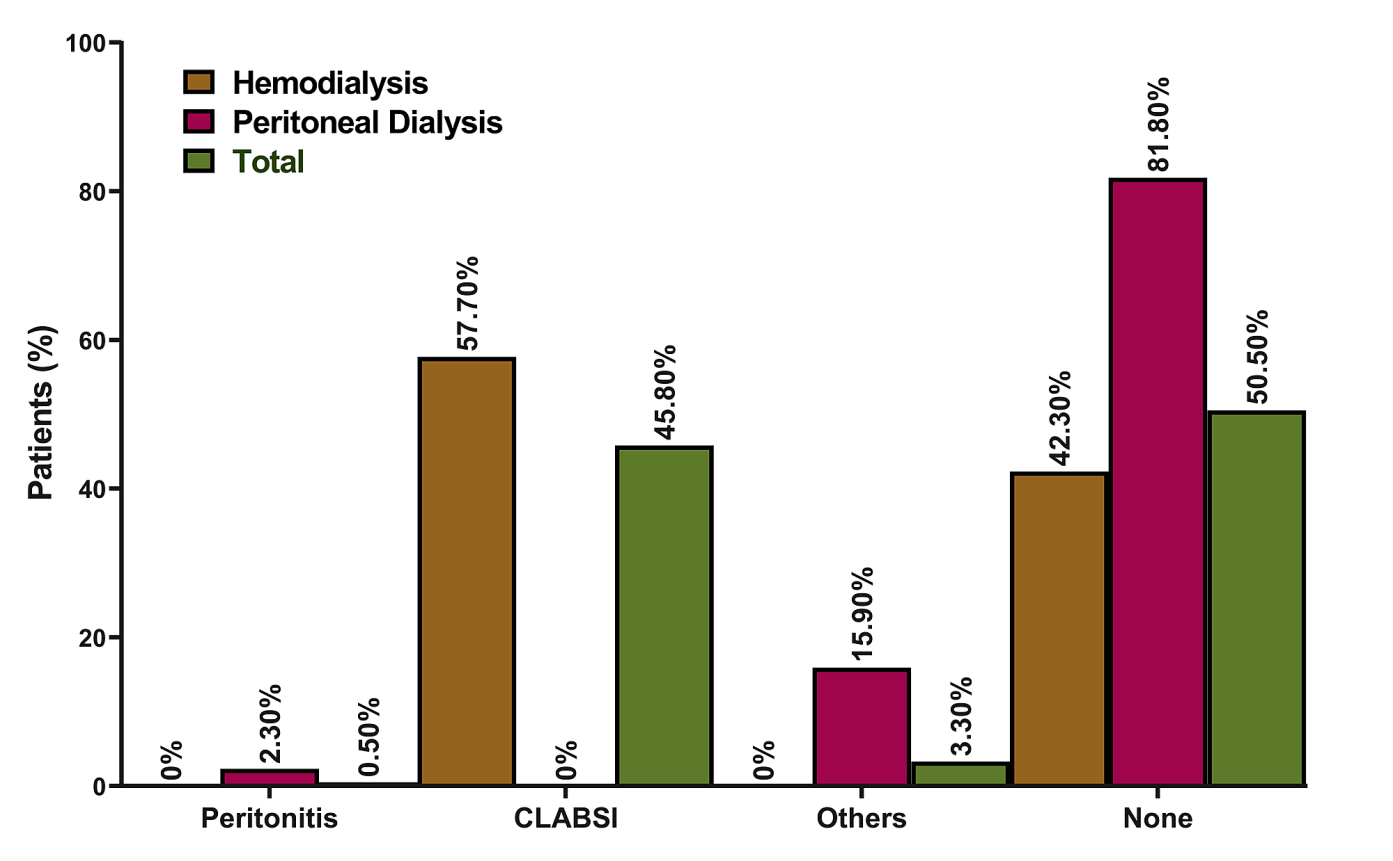
Distribution of infections encountered (according to the dialysis mode) CLABSI: central line-associated bloodstream infection

Hemodialysis patients showed longer mean admission days (3.7±3.8) as compared to PD patients (2.4±1.6). Similarly, the frequency of emergency visits was higher in HD patients (16.9±14.2) as compared to their peers with PD (14.4±12.6). Moreover, there was a significant increase in admission days for patients undergoing HD as compared to those having PD [OR (95% CI) = 1.27 (1.07-1.51); P-value = 0.007] (Table 3). On one hand, infection rates were higher among the HD group, where 57.70% of the patients had a central line-associated bloodstream infection (CLABSI). On the other hand, 81.80% of the PD group did not have any type of infections while 15.90% had other infections and 2.30% had peritonitis (Figure 2).

**Table 3:** Impact of dialysis time on patients’ outcomes CI: confidence interval, OR: odds ratio, SD: standard deviation; * statistically significant; ¶ Effect of dialysis type (hemodialysis) on different patients’ outcomes, where the data were adjusted for patients age, gender, socioeconomic status, and education

Variables	Mode of dialysis				Multivariate logistic regression¶	
	Hemodialysis		Peritoneal dialysis		OR (95 CI)	P-value
	Mean	SD	Mean	SD		
Admission (days)	3.7	3.8	2.4	1.6	1.27 (1.07-1.51)	0.007*
Emergency visits	16.9	14.2	14.4	12.6	1.01 (0.99-1.04)	0.397

## Discussion

PDEP elucidates the overall picture of PD and simplifies the better outcomes that could be obtained from conducting PD over HD. In our study, 75 ESRD patients received a PDEP. Of these patients, 42.7% of patients preferred PD, with an estimated negative statistical significance of PDEP on conducting HD. Kutner et al. reported that only 10.9% of the patients responded to the PDEP program, which is lower than our reported rate [23]. The authors justified this low rate by the shortage of content and duration information regarding PD or that the patients were less satisfied with the process. The authors of the same study also did not find any significance in discussing PD with patients 12 months before initiating regular treatment on preferring PD over HD. On the other hand, almost all previous studies have reported that pre-dialysis education impacted the overall rates of preferring PD and self-care. Ribitsch et al. conducted a case-control study and used a PDEP program as the intervention for the case group while the control did not receive any education [24]. The authors reported that 54.3% of the patients in the case group chose to initiate their RRT with PD while only 28% of patients in the control group preferred the same modality. The authors, however, excluded patients with emergencies and those referred to the center at a late stage. The importance of performing PD and self-care has been indicated with similar studies that have also reported similar rates. In a big study conducted by the National Pre-ESRD Education Initiative, which enrolled 15,000 ESRD patients and provided them with PDEP, the results showed that 45% of these patients preferred PD while the rest preferred HD [25]. The same rate was also reported by Little et al., which investigated the results of 254 patients [26]. Additionally, Manns et al. conducted a randomized trial and found that 82.1% in the intervention group chose PD, which was significant than the standard care group [27].

The rate of preferring PD over HD after PDEP is dependent on many factors. Our analysis results showed that the PDEP and non-PDEP patients’ age was statistically non-significant. However, Ribitsch et al. reported that initiating RRT was much observed among younger patients with fewer comorbidities and more social activities while older patients preferred HD due to more morbidities and more spare time to regularly visit the dialysis centers [24]. Therefore, every group of patients would choose the most suitable modality that meets their needs and makes them comfortable [28]. Additionally, the early initiation of the PDEP program, while CKD patients are still stable, has a positive outcome on the preference of self-care modalities over the center-based ones together with other outcomes as the frequency of hospitalization and the deterioration level of the case with regards to ESRD [26]. Moreover, using PD as the only self-care modality might be a limitation for many patients, and therefore, other self-care approaches should be provided and discussed in the PDEP programs [29]. Another reason is that such educational programs provide equal insights about the two modalities and some patients are more satisfied with their current regimens than going through changes and future experiments [29]. Finally, we also noticed that CLABSI was a significant factor in preferring HD over PD compared to other causes, which were associated with preferring PD over HD.

Our analysis results showed that PDEP was significantly associated with lower HD rates. Moreover, favorable outcomes, including reduced mean admission days and emergency visits in patients contributed to preferring PD over HD. Many studies have also reported that PD has favorable outcomes, as the fewer overall costs improve the QoL, and the increased patients’ satisfaction with PD despite showing that PD and HD were similar in terms of infections and complications [30]. Therefore, we recommend that PDEP programs should be simpler by delivering more persuasive information about the advantages of self-care and PD over center-based HD.

Missing data from some patients’ profiles might have been the only limitation in our study. However, whenever data was missing, patients were contacted by telephone or by recalling for a direct interview, and most of the missing information was filled.

## Conclusions

PDEP might have a positive impact on patients preferring PD over HD. Moreover, patients with infections and increased hospital and emergency admission periods were more likely to prefer HD over PD. The value of educational programs among dialysis patients is underestimated and has a very strong impact on their lives when done in a constructive and informative way.
